# The Association between Polyclonal Combined Serum Free Light Chain Concentration and Mortality in Individuals with Early Chronic Kidney Disease

**DOI:** 10.1371/journal.pone.0129980

**Published:** 2015-07-01

**Authors:** Lakhvir K. Assi, Natasha McIntyre, Simon Fraser, Scott Harris, Colin A. Hutchison, Chris W. McIntyre, Paul Cockwell, Maarten W. Taal

**Affiliations:** 1 The Binding Site Group Ltd, 8 Calthorpe Road, Edgbaston, Birmingham, United Kingdom; 2 Division of Medical Sciences and Graduate-Entry Medicine, University of Nottingham, Nottingham, United Kingdom; 3 Academic Unit of Primary Care and Population Sciences, University of Southampton, Southampton, United Kingdom; 4 Hawke’s Bay District Health Board, Hawke’s Bay, New Zealand; 5 Renal Unit, Royal Derby Hospital, Derby, United Kingdom; 6 Department of Renal Medicine, Queen Elizabeth Hospital Birmingham, Birmingham, United Kingdom; 7 Division of Immunity and Infection, College of Medical and Dental Sciences, University of Birmingham, Birmingham, United Kingdom; University of Leicester, UNITED KINGDOM

## Abstract

A major component of increased mortality risk in people with chronic kidney disease (CKD) is associated with non-traditional cardiovascular risk factors including markers of inflammation. We studied whether a novel marker of systemic inflammation, elevated serum combined polyclonal immunoglobulin free light chains (cFLC), was an independent risk factor for increased all-cause mortality in people with CKD stage 3. In a prospective community based cohort study, 1695 participants with stage 3 CKD and no cases of monoclonal gammopathy had cFLC concentrations measured. cFLC levels were determined using the summation of Freelite kappa and lambda assays. All other bioclinical variables were collected at the time of sample collection. Kaplan-Meier plots and Cox proportional hazards analysis was used to assess the relationship between high cFLC levels (>43.3 mg/L) and mortality. There were 167 deaths (10%) after a median of 1375 days. cFLC levels at recruitment were higher in participants who died compared with those who were alive at the end of the study; median: 46.5 mg/L (IQR: 36.1-65.4 mg/L) and 35.4 mg/L (28.1-46.6 mg/L) respectively, P <0.001. Kaplan-Meier survival analysis demonstrated participants with cFLC >43.3 mg/L levels had an increased risk of mortality compared to people with normal cFLC levels (P <0.001). Elevated cFLC levels were independently associated with worse survival (Hazard ratio: 1.50; 95% confidence interval: 1.04-2.16; P=0.03). Other independent risk factors for worse survival were: older age, male gender, previous cardiovascular event, lower eGFR and higher high sensitivity C-reactive protein (hsCRP). To conclude, high cFLC levels predict increased mortality in people with stage 3 CKD, independent of established risk factors and other markers of inflammation.

## Introduction

Chronic kidney disease (CKD) is associated with an increased risk of mortality that increases as glomerular filtration rate (GFR) decreases below 60 ml/min per 1.73m^2^ [1, 2]. Whilst some of the increased mortality risk is associated with traditional cardiovascular risk factors, including hypertension, diabetes and dyslipidaemia, a major component is attributable to non-traditional factors [3, 4]. Among these, systemic inflammation is emerging as an important contributor to the pathogenesis of cardiovascular disease (CVD) associated with CKD. Previous studies have focused on C-reactive protein (CRP) or cytokines as markers of inflammation [5, 6] but alternative biomarkers are needed to further improve detection of a subclinical inflammatory state and facilitate risk prediction in individuals with CKD.

One novel biomarker of systemic inflammation is serum free light chains (FLC). Each cell of the B-cell lineage produces one of two isotypes of immunoglobulin (Ig) light chain (LC), kappa (κ) or lambda (λ). The majority of Ig LC produced is incorporated into intact Ig molecules, but around 500 mg/day is released into the extracellular compartment [7, 8]. At molecular weights of ~22.5 kDa (κFLC) and ~45 kDa (λFLC), these molecules are predominantly cleared by the kidneys and therefore accumulate with declining kidney function [9, 10]. In addition, elevated polyclonal FLC levels occur with global immune activation [11, 12] and may therefore serve as a marker of systemic inflammation. Moreover, recent studies have shown that elevated combined polyclonal FLC levels (cFLC = κFLC plus λFLC) are associated with increased mortality in both community based and secondary care non-CKD cohorts [13–15].

We have previously shown a weak independent association between λFLC levels and mortality in a cohort of people with advanced CKD (median eGFR 21.9 ml/min per 1.73m^2^) and high vascular comorbidity recruited from secondary care clinics [16] and a stronger association was seen between cFLC and mortality in a larger secondary care cohort incorporating all non-dialysis CKD stages [17]. However there has been no assessment of the relationship between cFLC and mortality in early CKD when the patient would still be under the care of a general practitioner, and when risk stratification is arguably even more important. Therefore, the purpose of this study was to assess the relationship between cFLC levels and mortality in a cohort of people with early CKD, predominantly stage 3. Elevated cFLC was independently associated with poorer overall survival within this population.

## Materials and Methods

**Fig 1:**
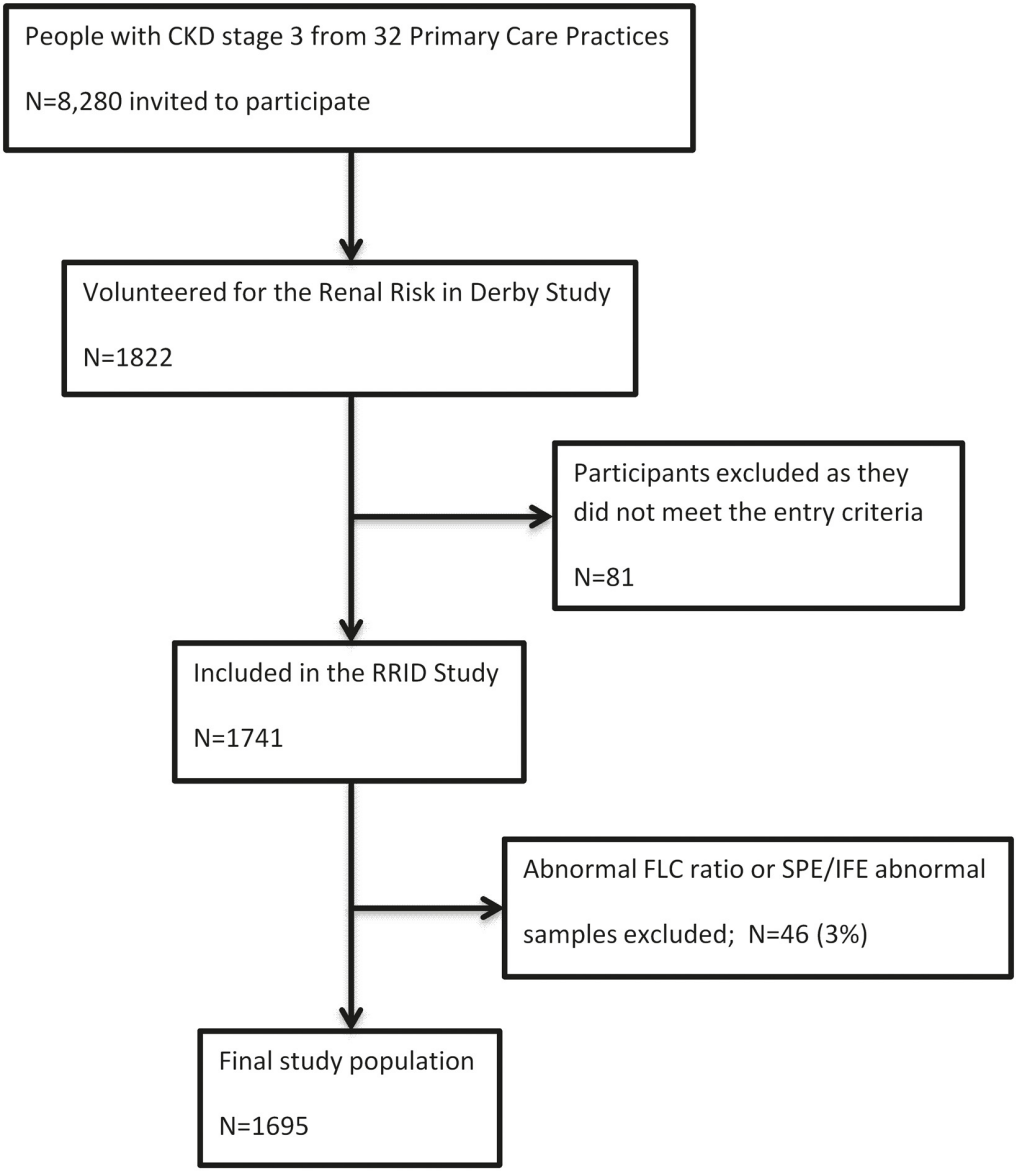
Study flow diagram.

The Renal Risk in Derby (R^2^ID) study is a large prospective cohort study to investigate outcomes in 1741 patients with early CKD under follow-up by a primary care physician. Participants were recruited directly from 32 community (primary care) medical centres on the basis of two previous eGFR values of 30–59 ml/min per 1.73m^2^ at least 3 months apart. Due to intra-patient variability in the eGFR test, some patients had an eGFR value outside the stage 3 CKD range at the first study visit. These patients were included in the study because they met the entry criteria prior to the first study visit. First study sample collections were conducted between August 2008 to March 2010 and last follow-up was set to 24^th^ February 2014 (Fig 1). The study collected a detailed bioclinical dataset including serum samples that were aliquoted and stored at -80°C for future analysis. Detailed methods have been published previously [18–20]. The cohort is under long-term follow-up for clinical outcomes including mortality.

Forty-six participants (3%) recruited in the complete cohort were not included in this study as they had evidence of a monoclonal gammopathy as defined by the presence of a monoclonal band by serum protein electrophoresis (SPE) and immunofixation electrophoresis (IFE) (Sebia Hydragel Immunofixation PE kit on the Hydrasys system) and/or an abnormal FLC ratio, indicating clonal expression of FLC, as determined using the renal reference range (0.37–3.1) [9]. FLC values obtained in the study population were compared with values previously obtained in a group of normal volunteers (N = 282, age range: 21–90 years), using the same assay, details of which have been published previously [21].

The study was approved by the Nottingham Research Ethics Committee 1 and abides by the principles of the Declaration of Helsinki. All participants provided written consent. The study was included on the National Institute for Health Research (NIHR) Clinical Research Network Portfolio (NIHR Study ID: 6632) and was independently audited by QED Clinical Services in November 2009.

### Outcome data

Participants were registered with the UK Health and Social Care Information Service to obtain date and cause (as recorded on the death certificate) of all deaths. The cause of death was classified independently by three clinical investigators as cardiovascular, cancer, infection and other. Differences in classification were resolved through discussion to reach a consensus.

### Laboratory analyses

Samples were analysed for serum κFLC, serum λFLC [22] (Freelite, The Binding Site Group Ltd, Birmingham, UK) and serum high-sensitivity CRP (hsCRP, Roche Diagnostics, Newhaven, UK). Assays were performed using a Roche Modular P Analyser (Roche Diagnostics) and run in accordance with the manufacturer’s instructions. The normal reference ranges used for the FLC analyses were: κFLC 3.3–19.4 mg/L, λFLC 5.71–26.3 mg/L [21]. The cFLC concentration was calculated for each sample by addition of the κFLC and λFLC values. The published 95^th^ percentile normal range for cFLC is 9.3–43.3 mg/L [14].

The creatinine assay has been standardized against an isotope dilution mass spectrometry (IDMS) method and the 4-variable Modification of Diet in Renal Disease (MDRD) equation modified for use with IDMS standardized creatinine measurement was used to estimate GFR. This study was commenced prior to publication of the CKD-EPI formula and eGFR derived from the MDRD formula was therefore used for recruitment and baseline study assessments.

### Clinical outcomes and statistical analysis

Statistical differences between cFLC and other biomarkers on a continuous scale were assessed using Kruskall-Wallis or Mann Whitney U tests. Categorical variables were compared using Chi-squared analysis. Correlation analyses were performed using Spearman rank analysis.

Univariate and multivariable logistic regression analyses were used to identify the associations of elevated cFLC (>43.3 mg/L). The multivariable model included variables that were statistically significant (P <0.05) on univariate analyses (with the exception of total cholesterol/HDL ratio as cholesterol and HDL were included separately). Variables were also excluded from the multivariable model where there was no clear clinical rationale for their inclusion (sodium, potassium and urea).

The cohort was followed prospectively until death or the end of the study period. Patients who were alive at the end of the study were censored according to their follow up time. A Kaplan-Meier curve was generated to determine the frequency of death over the period of follow up. Log-rank analysis was used to calculate the significance. Univariate analysis was used to determine which factors were associated with time to mortality. Univariate comparisons were made with subsequent addition of variables to the multivariable Cox proportional hazards model if they were found to have P <0.10 on univariate analysis and were considered clinically relevant. The order of addition of variables was socio-demographic (age, gender) and then clinical or laboratory (CVD, diabetes, hypertension, smoking, BMI, central obesity, total:HDL cholesterol ratio, eGFR, uACR, hemoglobin (Hb), platelets, white blood cell count (WBC), cholesterol, HDL, uric acid, diastolic blood pressure (DBP). The primary outcome was all-cause mortality. Despite meeting the inclusion criteria (and therefore having a clinical diagnosis of CKD stage 3), some participants were found to have a baseline eGFR ≥60ml/min per 1.73m^2^. Sensitivity analyses were therefore conducted in only those whose baseline eGFR was <60ml/min per 1.73m^2^ (n = 1283). All statistical analysis was performed using SPSS v19.0 (SPSS Chicago, USA) and Stata v12.0 (StataCorp. 2011. Stata Statistical Software: Release 12. College Station, TX: StataCorp LP).

## Results

### Population characteristics and biochemical markers

The baseline demographic and laboratory data of the study population are shown in Tables 1 and 2. Subjects were predominantly white (97%) and female (61%), with a median age of 74 years (interquartile range (IQR): 67–79 years). 17% had diabetes and 22% had previous cardiovascular event (as defined by a self-reported previous cardiovascular event). The median eGFR was 53.2 ml/min per 1.73m^2^ (IQR: 45.6–59.8 ml/min per 1.73m^2^).

**Table 1:** Baseline patient demographics and clinical variables in relation to serum cFLC levels.

Variable	Whole CKD population	cFLC ≤43.3mg/L N = 1111	cFLC >43.3mg/L N = 584	P value
**Age** (Years)	74 (67–79)	73 (66–78)	76 (70–81)	<0.001
**Male** (%) **Female** (%)	39 61	33 67	53 47	<0.001
**Ethnicity (%)**				
*White*	97	98	95	
*Asian*	1.7	1	3	0.03
*African-Caribbean*	0.3	0.1	0.7	
*Other*	1	0.9	1.3	
**Smoker** (%)	5	4	6	0.05
**Total pack years**	0.8 (0–195)	0 (0–195)	5 (0–141)	<0.001
**Diabetic** (%)	17	14	23	<0.001
**Previous CVD** (%)	22	20	27	0.03
**BMI** (kg/m^2^)	28.6 (25.7–31.9)	28.6 (25.7–31.9)	28.5 (25.7–31.8)	0.70
**WHR**	0.9 (0.8–1.0)	0.9 (0.8–1.0)	0.9 (0.9–1.0)	<0.001
**PWV** (m/s)	9.8 (8.5–11.1)	9.6 (8.5–10.9)	10 (8.7–11.3)	0.01
**SBP** (mmHg)	133 (122.3–114.0)	133 (122.0–143.0)	134 (122.7–146.3)	0.07
**DBP** (mmHg)	73.0 (65.0–80.3)	73.7 (66.0–81.3)	70.3 (62.7–79.0)	<0.001

Categorical variables analysed using Chi-square test and continuous variables were analysed using independent Mann-Whitney U test. ACR = urinary albumin/creatinine ratio, BMI = body mass index, cFLC = combined free light chains, CVD = cardiovascular disease, DBP = diastolic blood pressure, PWV = pulse wave velocity, SBP = systolic blood pressure, WHR = waist hip ratio.

**Table 2:** Baseline patient characteristics in relation to serum cFLC levels.

Variable	Whole CKD population	cFLC ≤43.3mg/L N = 1111	cFLC >43.3mg/L N = 584	P value
**Urea** (mmol/L)	8 (6.6–9.9)	7.4 (6.3–8.7)	9.5 (7.8–11.5)	<0.001
**eGFR** (ml/min per 1.73m^2^)	53.2 (45.6–59.8)	56.0 (49.6–61.7)	46.2 (39.2–53.7)	<0.001
**Albumin** (g/L)	41 (39–43)	41 (39–43)	40 (38–42)	<0.001
**Potassium** (mmol/L)	4.4 (4.1–4.7)	4.3 (4.1–4.6)	4.5 (4.2–4.8)	<0.001
**Sodium** (mmol/L)	140 (138–141)	140 (138–142)	139 (138–141)	<0.001
**Bicarbonate** (mmol/L)	26 (24–27)	26 (24–27)	25 (23–27)	<0.001
**Total Protein** (g/L)	74 (71–77)	74 (71–77)	75 (71–78)	<0.001
**Cholesterol** (mmol/L)	4.6 (3.9–5.5)	4.8 (4.1–5.7)	4.3 (3.6–5.1)	<0.001
**HDL** (mmol/L)	1.4 (1.1–1.7)	1.5 (1.2–1.8)	1.3 (1–1.6)	<0.001
**Uric acid** (μmol/L)	379 (320–441)	360 (307–418)	415 (355–481)	<0.001
**Hb** (g/L)	133 (123–141)	134 (125–143)	128 (119–138)	<0.001
**Correted calcium** (mmol/L)	2.4 (2.3–2.4)	2.4 (2.3–2.4)	2.4 (2.3–2.4)	0.78
**Phosphate** (mmol/L)	1.1 (1–1.2)	1.1 (1–1.2)	1.1 (1–1.2)	0.18
**hsCRP** (mg/L)	2.23 (1.14–4.62)	2.02 (1.1–3.9)	2.97 (1.4–5.6)	<0.001
**ACR** (mg/mmol)	0.3 (0–1.5)	0.2 (0–0.8)	1.0 (0.1–4.1)	<0.001
**Platelet count** (x10^3^/μL)	239 (202–284)	239 (206–284)	237 (197–284)	0.59
**WBC** (x10^3^/μL)	6.9 (5.8–8.1)	6.7 (5.8–8.0)	7.1 (6.0–8.3)	<0.001
**Serum κFLC** (mg/L)	19.2 (14.4–25.5)	NA	NA	NA
**Serum λFLC** (mg/L)	17.3 (13.5–22.7)	NA	NA	NA
**κ/λ ratio**	1.1 (0.9–1.3)	NA	NA	NA
**cFLC** (mg/L)	36.3 (28.6–47.9)	NA	NA	NA

Categorical variables analysed using Chi-square test and continuous variables were analysed using independent Mann-Whitney U test. NA = not applicable, ACR = urinary albumin/creatinine ratio cFLC = combined free light chains, eGFR = estimated glomerular filtration rate (calculated using the Modification of Diet in Renal Disease (MDRD) equation), FLC = free light chains, Hb = hemoglobin, κ = kappa, HDL = high density lipoprotein, hsCRP = high sensitivity C-reactive protein, λ = lambda, WBC = white blood cell count.

### Polyclonal serum free light chains

**Fig 2:**
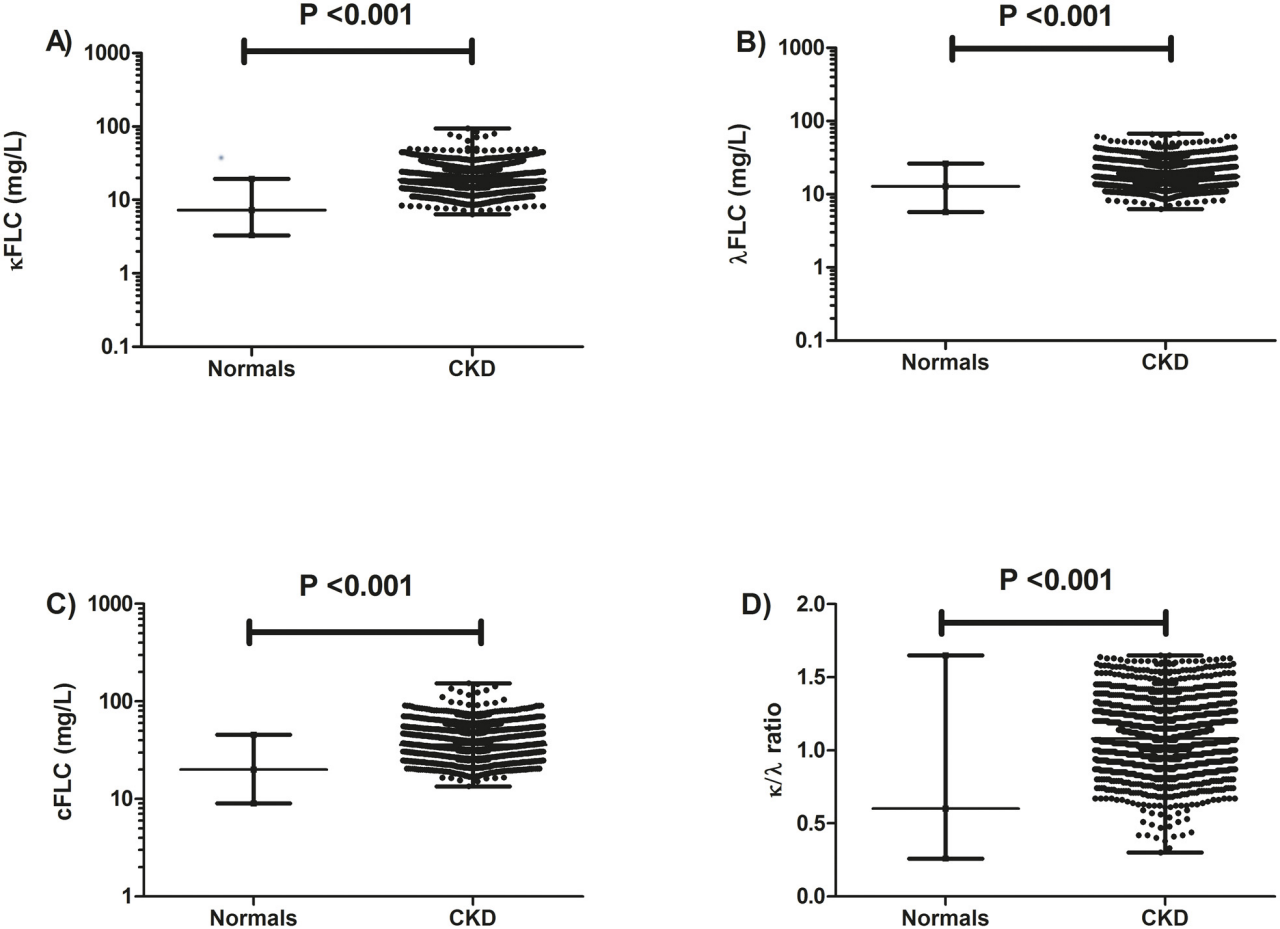
Serum FLC concentration in people with stage 3 chronic kidney disease versus normal controls. A) κfree light chains (FLC), B) λFLC and C) Combined free light chains (cFLC) levels were elevated in people with chronic kidney disease (CKD) (circles) versus published normal control individuals [21]. D) The FLC κ/λ ratio was also significantly higher in people with CKD compared to the healthy control population. Median and ranges are indicated (black bars).

**Fig 3:**
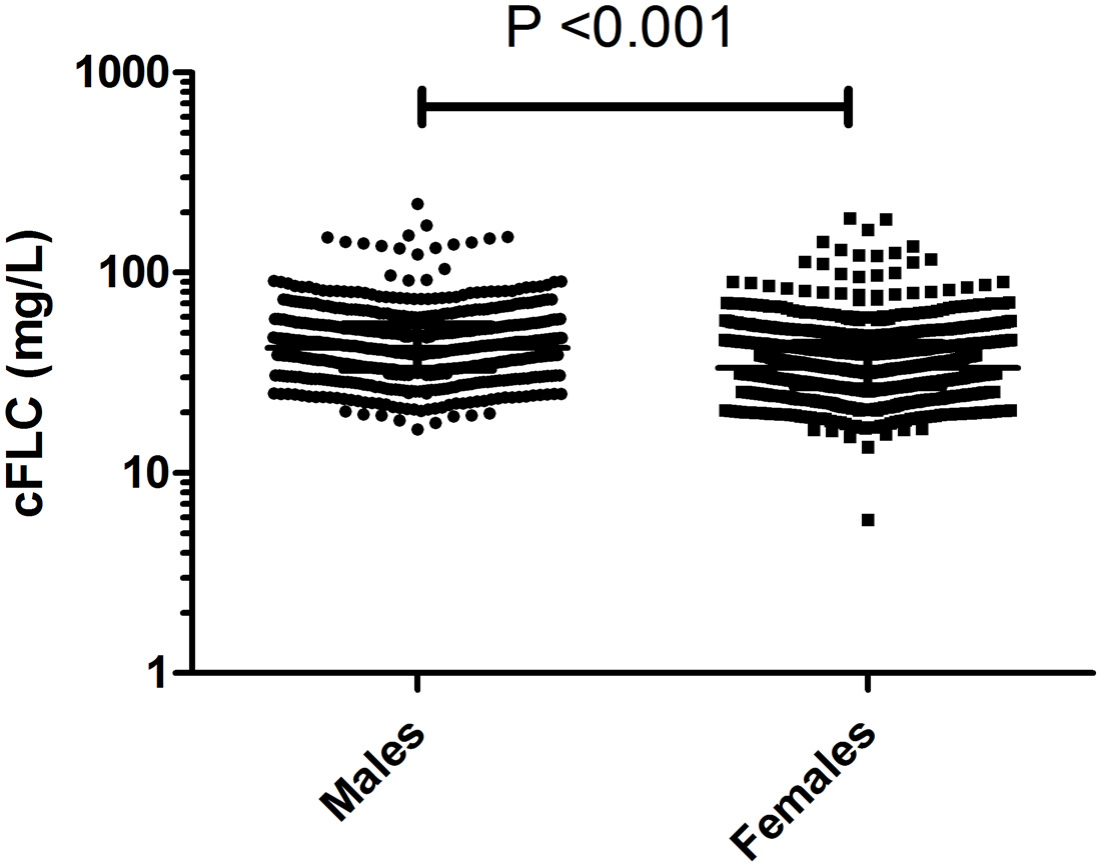
cFLC concentrations in males and females. cFLC concentrations were compared and were elevated in males compared to females (P<0.001). Median and interquartile ranges are indicated.

**Fig 4:**
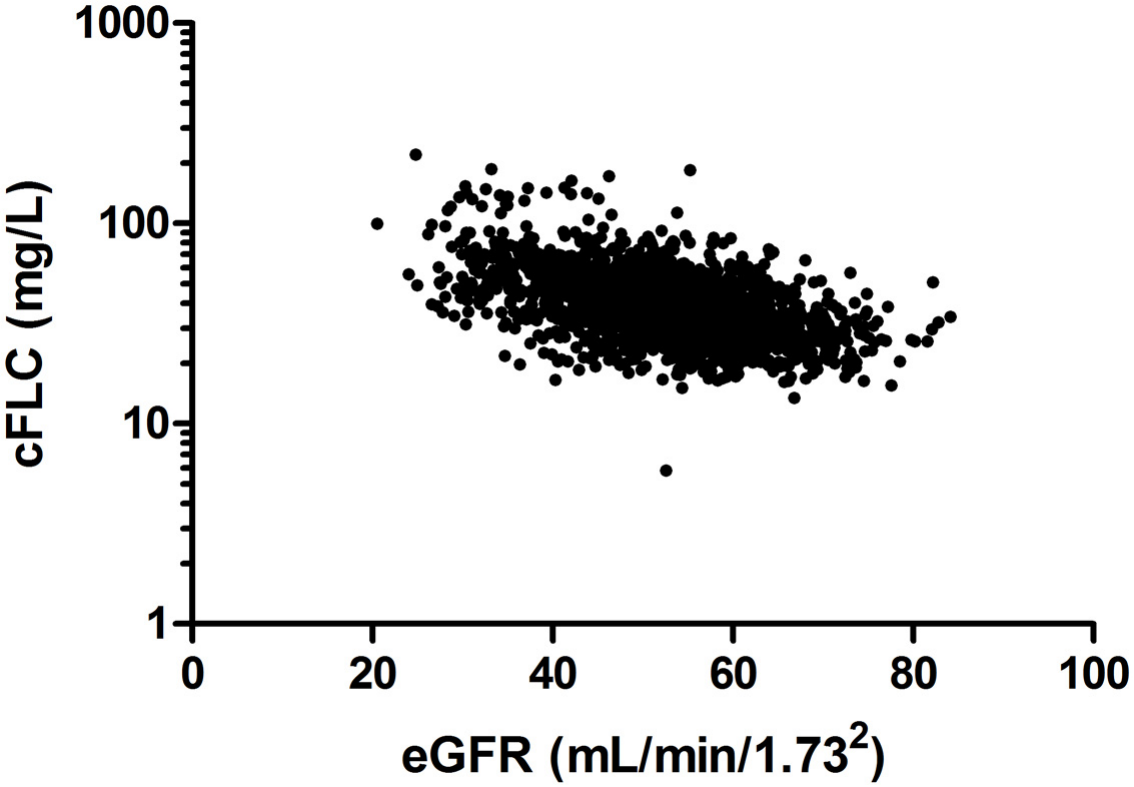
Correlation of cFLC and eGFR. There was a significant inverse association with cFLC (combined serum free light chains) and estimated glomerular filtration rate (eGFR) (rho = -0.49, P <0.0001). Spearman rank correlation was performed.

The median serum κFLC concentration was 19.2 mg/L (IQR: 14.4–25.5 mg/L) and serum λFLC concentration was 17.3 mg/L (IQR: 13.5–22.7 mg/L) (Table 2); these were significantly elevated compared to previously reported FLC values in a normal healthy control population [21, 23] (κFLC median 7.3 mg/L; λFLC median 12.4 mg/L, P <0.001) (Fig 2A and 2B). The median cFLC concentration for the CKD population was 36.3 mg/L (IQR: 28.6–47.9 mg/L) (Table 2 and Fig 2C); median κ/λFLC ratio was 1.1 (IQR: 0.9–1.3), which was significantly higher than controls, P <0.001 (Fig 2D). Males had significantly elevated cFLC concentrations compared to females: 41.95 mg/L (IQR:32.86–54.78 mg/L) vs 33.31 mg/L (IQR:26.85–44.32 mg/L) respectively P <0.001 (Fig 3). cFLC levels were elevated (>43.3 mg/L) in 584 (34%) patients. cFLC correlated inversely with eGFR (rho = -0.49, P <0.0001; Fig 4, Table 3). Weak but significant correlations were observed between cFLC and albumin (rho = -0.28), hsCRP (rho = 0.19), Hb (rho = -0.22), cholesterol (rho = -0.25), HDL cholesterol (rho = -0.23), PWV (rho = 0.11), uACR (rho = 0.32), DBP (rho = -0.14) and WHR (rho = 0.27) (P <0.01 for all). There was no significant correlation between cFLC and BMI, calcium or phosphate levels.

**Table 3:** Correlation of cFLC with other clinical markers.

Correlation of cFLC with:	Spearman rho	P value
Age	0.25	<0.001
Serum albumin	-0.28	<0.001
eGFR	-0.49	<0.001
hsCRP	0.19	<0.001
Haemoglobin	-0.22	<0.001
Cholesterol	-0.25	<0.001
HDL	-0.23	<0.001
Uric acid	0.32	<0.001
PWV	0.11	<0.001
ACR	0.32	<0.001
DBP	-0.14	<0.001
SBP	0.05	0.03
BMI	-0.03	0.20
WHR	0.27	<0.001
Calcium	-0.14	0.562
Phosphate	0.016	0.525

Correlation analyses (Spearman rho) were performed between cFLC and other clinical and laboratory markers in CKD. ACR = urinary albumin/creatinine ratio, BMI = body mass index, DBP = diastolic blood pressure, eGFR = estimated glomerular filtration rate, HDL = high density lipoprotein, hsCRP = high sensitivity C- reactive protein, PWV = pulse wave velocity, SBP = systolic blood pressure, WHR = waist hip ratio.

Participants with elevated cFLC (>43.3 mg/L) were older, had a higher waist to hip ratio (WHR), elevated pulse wave velocity (PWV), higher urinary albumin to creatinine ratio (uACR), elevated serum hsCRP and uric acid levels. Elevated cFLC was associated with lower levels of Hb, reduced albumin, and lower eGFR (Tables 1 and 2).

On univariate logistic regression analysis at baseline, elevated cFLC was associated with age, gender, diabetes, previous cardiovascular event, hypertension, smoking, eGFR, uACR, hsCRP, PWV, serum albumin, Hb, WBC, total protein, bicarbonate, cholesterol, HDL, and uric acid. The associations with age, gender, smoking, eGFR, uACR, serum albumin, Hb, total protein, cholesterol and HDL cholesterol were maintained after full adjustment (Table 4).

**Table 4:** Associations of elevated cFLC (>43.3 mg/L): univariate and multivariable logistic regression analyses.

Variable	Categories	Univariate OR (95% CI)	P value	Final multivariable model* OR (95% CI)	P value
**Age** (Years)	-	**1.05 (1.03–1.06)**	<0.001	**1.03 (1.01–1.05)**	0.004
**Gender** (vs female)	Male	**2.43 (1.97–2.98)**	<0.001	**1.88 (1.37–2.58)**	<0.001
**Diabetes** (vs no diabetes)	People with diabetes	**1.82 (1.40–2.35)**	<0.001	0.88 (0.62–1.25)	0.49
**CVD** (vs no CVD)	People with CVD	**1.50 (1.22–1.85)**	<0.001	0.86 (0.64–1.14)	0.28
**Hypertension** (vs no hypertension)	People with hypertension	**3.80 (2.04–7.09)**	<0.001	1.11 (0.70–1.75)	0.67
**Smoking** (vs never smokers)	Current smoker	**1.73 (1.08–2.78)**	<0.001	**2.13 (1.10–4.12)**	0.03
	Ex-smoker	**1.74 (1.41–2.15)**		**1.31 (0.99–1.72)**	
**eGFR** (ml/min per 1.73m^2^)		**0.91 (0.89–0.92)**	<0.001	**0.93 (0.91–0.94)**	<0.001
**ACR** ^**#**^		**1.49 (1.39–1.61)**	<0.001	**1.28 (1.17–1.40)**	<0.001
**High sensitivity CRP** ^**#**^ (mg/L)		**2.08 (1.68–2.59)**	<0.001	0.91 (0.67–1.24)	0.56
**PWV** (m/s)		**1.09 (1.04–1.15)**	0.001	0.95 (0.88–1.02)	0.14
**Serum albumin** (g/L)		**0.86 (0.83–0.89)**	<0.001	**0.77 (0.73–0.81)**	<0.001
**Hb** (g/dL)		**0.74 (0.69–0.80)**	<0.001	**0.80 (0.72–0.88)**	<0.001
**WBC** (x10^9^)		**1.07 (1.02–1.13)**	0.009	0.95 (0.89–1.02)	0.14
**Total protein** (g/L)		**1.07 (1.04–1.09)**	<0.001	**1.20 (1.16–1.24)**	<0.001
**Bicarbonate** (mmol/L)		**0.91 (0.88–0.95)**	<0.001	0.95 (0.91–1.00)	0.05
**Cholesterol** (mmol/L)		**0.71 (0.64–0.78)**	<0.001	**0.77 (0.67–0.88)**	<0.001
**HDL Cholesterol** (mmol/L)		**0.34 (0.26–0.44)**	<0.001	**0.63 (0.44–0.89)**	0.009
**Uric acid** (μmol/L)		**1.01 (1.01–1.01)**	<0.001	1.00 (1.00–1.00)	0.06
**Total:HDL cholesterol ratio**		**1.09 (1.00–1.18)**	0.05	**-**	-
**Central obesity** (vs not centrally obese)	People with central obesity	1.16 (0.87–1.54)	0.32	-	-
**BMI** (kg/m^2^)		1.00 (0.98–1.02)	0.63	**-**	-
**Platelet count** (x10^9^)		1.00 (1.00–1.00)	0.64	**-**	-
**Corrected calcium** (mmol/L)		0.85 (0.31–2.34)	0.76	**-**	-
**Phosphate** (mmol/L)		1.61 (0.92–2.85)	0.10	**-**	-

All variables are continuous except for gender, diabetes, previous cardiovascular event, hypertension, smoking status and central obesity.

^#^Log transformed variable.

*Adjusted for age, gender, diabetes, cardiovascular disease, hypertension, smoking, eGFR, uACR, hsCRP, pulse wave velocity, serum albumin, Hb, WBC, total protein, bicarbonate, cholesterol, HDL, and uric acid. OR = Odds ratio, 95% CI = 95% confidence interval. ACR = albumin/creatinine ratio, BMI = body mass index, CVD = cardiovascular disease, cFLC = combined free light chains, HDL = high density lipoprotein, hsCRP = high sensitivity C-reactive protein,, WBC = white blood cell count. Significant variables are highlighted in bold.

### Survival analysis

**Fig 5:**
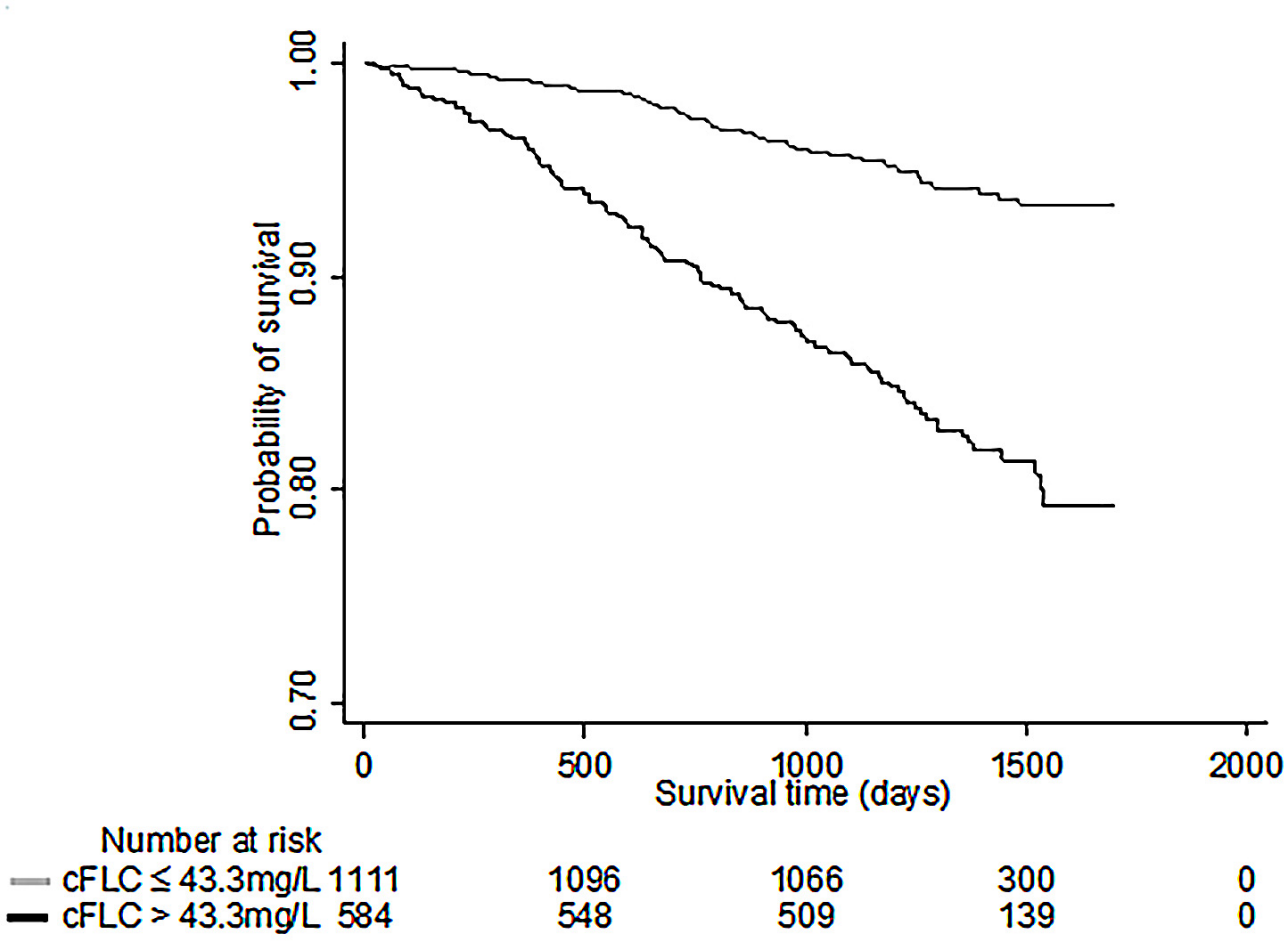
Kaplan-Meier plot of survival in people with elevated (>43.3mg/L) versus normal combined serum cFLC concentration. Participants with combined free light chains (cFLC) ≤43.3 mg/L had significantly longer overall survival compared to participants with cFLC >43.3 mg/L. Log rank analysis: P <0.001. Note that the *x* axis does not intersect the *y* axis at zero.

At analysis, the median duration of patient follow-up was 1375 days (1152–1502). There were 167/1695 deaths from all causes (10%). Of the total deaths, 102 patients (61%) had elevated cFLC. The most frequent primary causes of death were CVD (42%), infections (29%), cancer (21%) and death due to other causes (8%). The median cFLC concentration at recruitment was significantly higher in participants who died compared with those who were alive at the end of the observation period; 46.5 mg/L (IQR: 36.1–65.4 mg/L) vs 35.4 mg/L (IQR: 28.1–46.6 mg/L), P <0.001. Kaplan-Meier survival analysis demonstrated that patients with a cFLC level in the normal range (≤43.3 mg/L) had better overall survival than those with cFLC >43.3 mg/L (log-rank analysis, P <0.001) (Fig 5).

Univariate Cox proportional hazards analysis identified a number of factors that were significantly associated with higher all-cause mortality (Table 5). Multivariable analyses showed that elevated cFLC concentration was an independent predictor of all-cause mortality in addition to older age, gender, previous cardiovascular event, lower eGFR and higher hsCRP concentrations (Table 5). Sensitivity analysis including only patients with eGFR <60ml/min per 1.73m^2^ at baseline gave similar results although there was no longer an association with eGFR on multivariable analysis (HR: 0.99; 95% CI: 0.97–1.02); all other associations remained.

**Table 5:** Univariate and multivariable Cox proportional hazards analyses showing determinants of all-cause mortality.

Variable	Categories	Univariate HR (95% CI)	P value	Final multivariable model* HR (95% CI)	P value
**Age**	-	**1.10 (1.07–1.12)**	<0.001	**1.07(1.05–1.10)**	<0.001
**Gender** (vs female)	Male	**2.03 (1.50–2.76)**	<0.001	**1.44 (1.02–2.03)**	0.04
**Diabetes** (vs no diabetes)	People with diabetes	**1.52 (1.06–2.17)**	0.02	1.21 (0.82–1.78)	0.33
**CVD** (vs no CVD)	People with CVD	**3.28 (2.40–4.47)**	<0.001	**2.33 (1.68–3.23)**	<0.001
**Hypertension** (vs no hypertension)	People with hypertension	1.27 (0.77–2.09)	0.36	0.83 (0.48–1.44)	0.51
**Smoking** (vs never smokers)	Current smoker	**1.59 (0.79–3.21)**	0.004	1.99 (0.96–4.12)	0.16
	Ex-smoker	**1.73 (1.25–2.40)**		1.03 (0.72–1.46)	
**eGFR** (ml/min per 1.73m^2^)		**0.94 (0.93–0.96)**	0.06	**0.98 (0.96–0.99)**	0.003
**uACR** ^**#**^ (mg/mmol)		1.00 (1.00–1.01)	0.34	1.06 (0.94–1.19)	0.33
**High sensitivity CRP** ^**#**^ (mg/L)		**2.55 (1.90–3.41)**	<0.001	**1.83 (1.32–2.52)**	<0.001
**Central obesity** (vs not centrally obese)	People with central obesity	0.91 (0.60–1.37)	0.64	0.84 (0.55–1.28)	0.42
**PWV** (m/s)		**1.14 (1.06–1.23)**	<0.001	0.98 (0.90–1.07)	0.66
**Serum albumin** (g/L)		**0.90 (0.87–0.93)**	<0.001	0.96 (0.91–1.01)	0.11
**cFLC** (mg/L) (Categorical vs cFLC ≤43.3 mg/L)	People with cFLC >43.3 mg/L	**3.20 (2.34–4.36)**	<0.001	**1.50 (1.04–2.16)**	0.03
**BMI** (kg/m^2^)		0.97 (0.94–1.00)	0.06	**-**	-
**Total:HDL cholesterol ratio**		1.00 (0.89–1.14)	0.94	**-**	-
**Hb** (g/dL)		**0.81 (0.73–0.90)**	<0.001	**-**	-
**Platelet count** (x10^9^)		**0.99 (0.99–1.00)**	0.003	**-**	-
**WBC** (x10^9^)		**1.10 (1.05–1.16)**	<0.001	**-**	-
**Potassium** (mmol/L)		**1.55 (1.12–2.13)**	0.008	**-**	-
**Sodium** (mmol/L)		1.03 (0.98–1.09)	0.3	**-**	-
**Urea** (mmol/L)		**1.12 (1.09–1.15)**	<0.001	**-**	-
**Corrected calcium** (mmol/L)		0.3 (0.06–1.47)	0.14	**-**	-
**Phosphate** (mmol/L)		0.89 (0.37–2.11)	0.79	**-**	-
**Total protein** (g/L)		1.00 (0.97–1.03)	0.92	**-**	-
**Bicarbonate** (mmol/L)		1.01 (0.96–1.08)	0.63	**-**	-
**Cholesterol** (mmol/L)		**0.74 (0.64–0.86)**	<0.001	**-**	-
**HDL** (mmol/L)		**0.59 (0.40–0.86)**	0.007	**-**	-
**Uric acid** (μmol/L)		**1.00 (1.00–1.01)**	<0.001	**-**	-

All variables are continuous except for gender, diabetes, previous cardiovascular event, hypertension, smoking status and central obesity.

^#^Log transformed variable.

*Adjusted for age, gender, CVD, diabetes, hypertension, smoking, eGFR, albuminuria, hsCRP, central obesity, PWV, serum albumin. ACR = urinary albumin/creatinine ratio BMI = body mass index, cFLC = combined free light chains, CVD = cardiovascular disease, hsCRP = high sensitivity C-reactive protein, DBP = diastolic blood pressure, Hb = hemoglobin, HDL = high density lipoprotein, PWV = pulse wave velocity, SBP = systolic blood pressure,, WBC = white blood cell count, WHR = waist hip ratio. Significant variables are highlighted in bold.

## Discussion

Previous studies have reported an association between elevated FLC and increased risk of mortality in 2 independent cohorts of patients with advanced CKD attending specialist nephrology clinics in secondary care. Here, we have identified serum cFLC levels as an independent predictor of reduced survival in a large cohort of people with relatively early stage CKD, most of whom had never been referred to a nephrologist. Furthermore, this study represents the largest cohort of people with a known chronic disease that have been assessed for cFLC levels and mortality. Our observations therefore support the hypothesis that systemic inflammation is an important contributor to CVD and excess mortality in CKD. Moreover, our finding that cFLC was a predictor of reduced survival independent of hsCRP, suggests that cFLC is a more sensitive biomarker of systemic inflammation that adds prognostic information to hsCRP alone. We focused on stage 3 CKD because it includes the majority of patients with known CKD and is a heterogeneous group with variable prognosis in who improved risk stratification would be clinically useful. We chose survival as the primary outcome for this analysis because the major risk to these patients is decreased survival primarily due to CVD, rather than progression to end-stage kidney disease [24]. The prognostic value of cFLC is particularly important in this context because cardiovascular risk prediction tools developed for the general population do not take account of non-traditional risk factors and tend to underestimate risk in people with CKD [25]. Whilst the risk of adverse outcomes in the CKD 3 population is less than those patients with advanced disease, risk assessment of these patients remains of key importance. The CKD 3 population make up the majority of patients diagnosed with CKD, however many of these patients remain under the care of their general practitioner rather than a specialist nephrology team. Nevertheless, these patients have an increased risk of mortality (165% increased compared to a non-CKD population), hospitalisation due to cardiovascular complications (46% increased) and infection (42% increased) [26]. Therefore it is arguably more important to improve the stratification of these patients for risk of adverse outcomes so that their management can be adjusted accordingly.

We defined high cFLC concentrations as serum levels above the upper limit of the normal range in a healthy population [14]. We found that these levels were associated with an increased risk of death as assessed by Kaplan-Meier analysis and that cFLC remained independently associated with an increased risk of death by multivariate Cox proportional hazards analysis. Other independent variables associated with death included age, gender, eGFR, previous cardiovascular event and raised hsCRP. Thus, cFLC was a predictor of reduced survival independent of previously identified predictors.

A previous study that assayed for monoclonal proteins (including monoclonal FLC) and polyclonal FLC in patients with advanced CKD and high cardiovascular comorbidity showed a weak independent association between increasing λFLC levels and CKD-related outcomes; the study was conducted in a smaller secondary care population with advanced CKD (median eGFR 21.9 ml/min per 1.73m^2^) and a high prevalence of pre-existing CVD [16]. Markers of cardiac injury were also included in this study and these independently associated with mortality [16]. A recent study of 848 patients recruited from nephrology clinics with all stages of non-dialysis CKD (median eGFR 45 ml/min per 1.73m^2^; median urine ACR 10 mg/mmol) showed a stronger association when cardiac biomarkers were not included [17]. For comparison, the median eGFR in our primary care population was 53 ml/min per 1.73m^2^and median urine ACR was 0.3 mg/mmol. Our data therefore confirm and extend these observations by showing that serum cFLC concentration is a risk factor for reduced survival also in the early stages of CKD.

The rationale for using cFLC to aid stratification of CKD is twofold. Firstly, FLC represent a sensitive marker of kidney function, with an exponential relationship between their serum levels and eGFR [9]. This reflects the renal clearance of the molecules; individuals with normal kidney function typically clear κFLC in 2–4 hours at approximately 40% of the glomerular filtration rate and λFLC are cleared inbetween 3–6 hours at 20% of the glomerular filtration rate [27]. The secondary route of clearance for FLC is through the reticulo-endothelial system (RES). With end stage kidney disease and dominant clearance by the RES, the half-life of FLC extends from 2–6 hours to several days [8].

Secondly, polyclonal FLC represent a dynamic marker of the adaptive immune system [11, 28] as the clearance of the molecules is far more rapid than intact immunoglobulins; IgG has a half-life of around 21 days, IgA and IgM have half-lives of approximately 5–6 days [29, 30]. There are now several publications which describe the relationship between cFLC levels and disease activity in inflammatory diseases [11, 31–33], infections [15, 34, 35] and malignancies [36]. Moreover, inflammation is increasingly recognized as an important factor in the pathogenesis of CVD, with or without CKD. Recently, two studies have reported a relationship between elevated cFLC levels and mortality in general populations. Dispenzieri and colleagues showed that for each higher decile of cFLC, there was an increased risk of mortality in over 15,000 people from Olmsted County [14]. Similarly, in a study of individuals who had FLC measured to screen for a monoclonal disease but had no evidence of monoclonality, elevated cFLC was an independent risk factor for death [13]. Interestingly in our study, higher cFLC concentration was associated with reduced survival independent of hsCRP, suggesting that cFLC may be a more sensitive biomarker of systemic inflammation that adds prognostic information to hsCRP alone.

Whether elevated cFLC levels are just a marker of poor outcome or have a pathogenic role in disease is uncertain. There is evidence that cFLC can inhibit the phagocytic activity of leukocytes [37] and in autoimmune diseases activate mast cells [38]. However, there are no definitive mechanisms as yet for these effects and further work is ongoing to elucidate mechanisms whereby cFLC could contribute to cellular dysfunction in a range of settings.

Our study has several limitations. Although the cohort is broadly representative of people with CKD being managed in primary care in England [39], the study population was predominantly white and elderly. Our findings may therefore not be applicable to populations with a more diverse ethnic composition or in younger patients. Further research in other populations is required to confirm our findings. Second, further research with longer follow-up and more outcome events is required to identify whether serum cFLC adds significantly to the power of previously described risk factors such as eGFR and albuminuria to predict survival in people with CKD.

In conclusion, the relationship observed between elevated polyclonal cFLC levels and subsequent mortality risk in a population with early CKD adds further evidence to a role for cFLC as an independent risk marker. This may have particular relevance in the setting of CKD because risk prediction tools developed for the general population do not take account of non-traditional risk factors and tend to underestimate risk in people with CKD. One advantage of using cFLC as a biomarker is that, unlike many others, the assay for FLC is already widely available in routine clinical laboratories and clinicians are familiar with it as a common test to screen for monoclonal gammopathies. Further work is now required to determine if the prospective measurement of cFLC in CKD can be used to improve risk stratification in people with CKD and direct interventions to those at highest risk while sparing those at low risk from unnecessary treatment.
