# Correction: The Association between Polyclonal Combined Serum Free Light Chain Concentration and Mortality in Individuals with Early Chronic Kidney Disease

**DOI:** 10.1371/journal.pone.0141404

**Published:** 2015-10-20

**Authors:** Lakhvir K. Assi, Natasha McIntyre, Simon Fraser, Scott Harris, Colin A. Hutchison, Chris W. McIntyre, Paul Cockwell, Maarten W. Taal

There is an error in the units in Fig 4, “Correlation of cFLC and eGFR.” Please see the corrected Fig 4 here.

**Fig 4:**
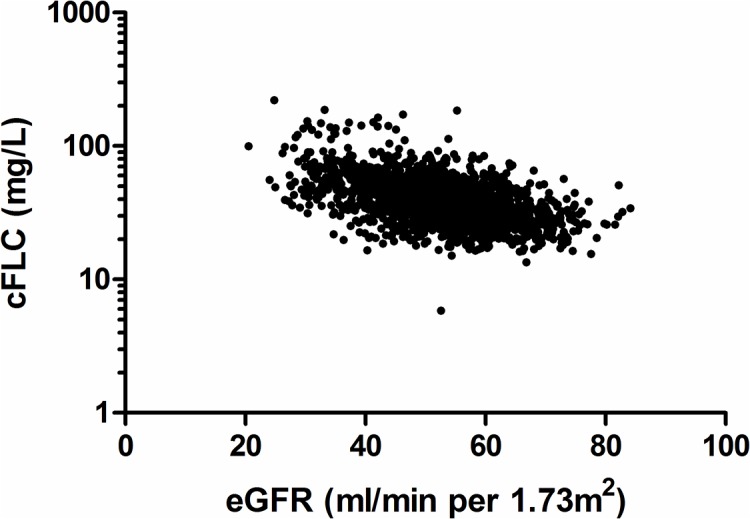
Correlation of cFLC and eGFR. There was a significant inverse association with cFLC (combined serum free light chains) and estimated glomerular filtration rate (eGFR) (rho = -0.49, P <0.0001). Spearman rank correlation was performed.
